# Mental health disparities in Latinx immigrant communities residing in the United States during COVID-19: Implications for policy and practice

**DOI:** 10.3389/fpubh.2022.1000233

**Published:** 2022-09-29

**Authors:** María Pineros-Leano, Nancy Jacquelyn Pérez-Flores, Katherine Damian, Kelli Rodrigues, Gabi Ortiz, Shannon D. Simonovich

**Affiliations:** ^1^MACONDO Research Team, School of Social Work, Boston College, Chestnut Hill, MA, United States; ^2^School of Social Work, Boston College, Chestnut Hill, MA, United States; ^3^Brown School of Social Work, Washington University in St. Louis, St. Louis, MO, United States; ^4^Morrissey College of Arts and Sciences, Boston College, Chestnut Hill, MA, United States; ^5^The Maternal Child Health Initiative, School of Nursing, College of Science and Health, DePaul University, Chicago, IL, United States

**Keywords:** COVID-19 pandemic, Latinx immigrant families, mental health, providers, qualitative research and analysis

## Abstract

**Objectives:**

Studies have demonstrated that Latinx populations face significant health disparities in access to mental health care. The objective of this study was to describe the impact of the COVID-19 pandemic on the mental health needs of Latinx families, from the perspectives of direct service providers working with Latinx communities.

**Methods:**

Twenty-one semi-structured interviews were conducted virtually with direct service providers to the Latinx community from August to October 2020. Interviews were transcribed verbatim and analyzed using thematic analysis.

**Results:**

Two-thirds of providers were female, with a median age of 33 years, and provided direct services to Latinx clients and had extensive experience working with immigrant families, particularly in Massachusetts. Key themes identified describing the impact of COVID-19 on the mental health needs of Latinx families included: (1) exacerbation of mental health symptoms, (2) financial stressors, (3) preoccupation regarding transnational lives, (4) secondary needs becoming more salient, and (5) immigration status as a main driver of inequality.

**Conclusions:**

Our findings highlight the importance of focusing on the mental health needs of Latinx immigrants and ensuring their access to mental health services. Telehealth seems to be a potential tool that promotes mental health access among Latinx clients. Future research needs to continue investigating the role of telehealth in decreasing mental health access disparities.

## Introduction

The Latinx population has a substantial presence throughout much of the U.S. (1). They constitute 60 million individuals, or 18.7% of the total U.S. population (2), and 50% of the foreign-born population in the U.S. (3, 4). Latinxs remain one of the country's largest ethnic minority groups, and their numbers are expected to grow due to continued immigration and higher fertility rates than Black and White populations (3).

Despite the growth of the Latinx population, the health and mental health infrastructure to address their needs is still lacking (5). Salsberg et al. found that Latinx providers are the most underrepresented ethnoracial group in healthcare (5). This lack of representation has direct implications on the care provided to the Latinx populations, which has been evidenced throughout the coronavirus disease 2019 (COVID-19) pandemic. The COVID-19 pandemic has disproportionately impacted Indigenous, Black, and Latinx individuals' physical health and mental health (6–8). The circumstances brought about by the pandemic have also resulted in additional barriers to health and mental health care, primarily due to, but not limited to, widespread loss of employment (9), the inadequate practice of social distancing due to living situations—multigenerational families or low-income and public housing- and high exposure to the virus as essential workers (9). Compared to other racial and ethnic groups in the US, the COVID-19 pandemic has negatively impacted the wellbeing of Latinx communities (6, 7). Latinx individuals in the US were 1.7 times more likely to contract COVID-19, 4.1 times more likely to be hospitalized, and 2.8 times more likely to die from COVID-19 complications in comparison to White individuals (10). In Massachusetts specifically, where this study was conducted, the Latinx population accounts for 12% of the total state population. At the beginning of the COVID-19 pandemic, Massachusetts was the state with one of the highest COVID-19 incidence rates (11). Similar to national findings, a study from Massachusetts indicated that majority-minority neighborhoods were highly impacted with Black and Latinx populations having the highest incidence rates of COVID-19 (11). Importantly, the study also found that the strongest predictor of COVID-19 cases was nativity; foreign-born populations had the highest incidence (11).

COVID-19 has also exacerbated disparities in mental health care access and utilization among Latinx communities, a disparity well-documented in the US even before the onset of the pandemic (12–16). These disparities are worsened by several barriers, including inadequate health insurance coverage and avoidance of government assistance programs, such as food and housing, due to a fear of deportation or legal ramifications (16–19).

Research describing the impact of the COVID-19 pandemic on the US-based Latinx population has been limited. Behbahani et al. (20) described Latinx individuals' difficulty accessing COVID-19 testing sites and treatment services. Previous to the pandemic, there were several reports of barriers to health and social services for Latinx individuals including lack of accessibility, availability, and affordability (21–23) in addition to institutional barriers such as lack of Spanish speaking healthcare team members that prevented Spanish-speaking Latinx clients from accessing services (22). However, these previous findings around barriers to health and social services for the Latinx communities in the US have not been examined during the COVID-19 pandemic. To date, there is a lack of research investigating how the mental health needs and barriers changed during the COVID-19 pandemic among Latinx immigrant families.

Given that providers are in constant communication with their clients, they can offer a unique perspective of clients' needs and how they changed throughout the COVID-19 pandemic. Furthermore, providers can offer essential information about the institutional practices and how these may have been affected during the unprecedented time of the pandemic (22). Despite the literature available on this topic, there is a lack of research investigating whether the mental health needs and barriers changed during the COVID-19 pandemic, particularly among Latinx immigrant families.

To address the gaps in the literature, this study was designed to better understand the mental health barriers that Latinx immigrants faced during the COVID-19 pandemic through the perspectives of direct service providers who work with the Latinx population. We interviewed predominantly mental health providers since they have direct experience with the Latinx population and understand their mental health needs. Specifically, the question that guided this study was: *What are the providers' perspectives on the mental health needs of Latinx immigrant families during the COVID-19 pandemic?*

## Materials and methods

This study utilized qualitative descriptive methodology to conduct semi-structured individual interviews with direct service providers to the Latinx community.

### Sample

Prospective study participants were recruited from 14 Latinx-serving community organizations, ranging from community health centers to middle schools, predominantly located in Massachusetts, United States. These community organizations were selected based on established relationships that the principal investigator and other collaborators already had with them. The research team met with representatives of several of these organizations and invited them to help in the recruitment process. The community organizations sent recruitment materials and study information to their teams. Prospective study participants were encouraged to contact the research team *via* email or phone. Inclusion criteria for participation included: (1) being a provider (e.g., health, mental health), (2) working primarily with the Latinx community, and (3) agreeing to engage in a 90-min interview. Exclusion criteria included providers who declined to be audio-recorded. Each prospective study participant was screened for eligibility before proceeding. Participants were given the option of completing the interview in English or Spanish.

### Data collection

Data collection was completed remotely from the privacy of participants' homes. All interviews were held *via* distance utilizing computer-based audio call services or telephone. Interviews were conducted by KR and KD, research assistants trained by the study principal investigator, MPL. Following formal data collection, team debriefing meetings were held to address any questions or issues that arose during the interview process. Before initiating the formal interview protocol, the research team member read the information sheet verbatim and obtained verbal consent from the participant. The audio-recorded interview began with a demographic questionnaire, followed by an in-depth interview that lasted an average of 77 min, with a minimum of 38 min and a maximum of 129 min. Twenty of the interviews were conducted in English. One interview was conducted in Spanish. The interview included questions related to the type of work the provider conducted with Latinx immigrants, the wellbeing of Latinx immigrant families, and how these families' needs were altered during the pandemic. Sample questions include: “*How do you feel the pandemic has affected Latinx immigrants?”* and “*Has your organization continued to provide all services since the pandemic began?”* (see Supplementary material for the full interview guide). Once the interviewers noticed that there were no new topics being described by the participants, we identified that data saturation had been reached and data collection was stopped once any remaining participants who had been scheduled were interviewed. The participants were compensated with a $40 gift card for their time. The Boston College Institutional Review Board authorized all procedures under protocol number 21.040.01e. All participants provided informed consent prior to beginning any research activities.

### Data analysis

Data were collected from direct service providers to the Latinx community utilizing semi-structured, in-depth interviews. Audio-recordings of the completed interviews were transcribed verbatim using a transcription provider. Each transcript was verified for accuracy by KD. Using thematic network analysis, the researchers used a grounded theory approach to allow the themes to emerge from the data (24). First, two coders went through all the interviews and assigned generic codes. The interview in Spanish was coded in Spanish to avoid losing any meaning in translation. Second, a codebook was developed based upon these generic codes. After creating the codebook, two coders (KD & KR) recoded all the interviews and identified illustrative quotes for each code. Each code had at least an 80% intercoder agreement. When there was <80% agreement, the coders discussed their coding decisions until general consensus was formed. Overall, the average intercoder reliability agreement among final codes was 93.1%. These final codes were grouped into key themes with coinciding illustrative quotes. Any quotes in Spanish were then translated to English.

## Results

Twenty-one in-depth interviews were conducted from August through October 2020. All participants had either a master's degree (*n* = 18; 87.5%) or a doctoral degree (*n* =3; 12.5%). Most participants provided mental health services (*n* = 16; 76%), three were case managers and two worked at schools. Two-thirds of providers were female, with a median age of 33 years. Fourteen providers (66.7%) identified as Latinx. Most participants (57%) had been practicing for 5 years or more. Providers worked predominantly with Latinx immigrant families from the Dominican Republic, El Salvador, and Guatemala.

### Themes

Five themes that impacted the mental health of US-based Latinx families during the COVID-19 pandemic were identified: (1) exacerbation of mental health symptoms, (2) financial stressors, (3) preoccupation regarding transnational lives, (4) secondary needs becoming more salient, (5) immigration status as a primary driver of inequality (see Table 1).

**Table 1:** Key themes and illustrative quotes.

**Exacerbation of mental health symptoms (*****n*** **=** **19; 90%)**
”… I just think that it's exacerbated symptoms of depression and anxiety… [COVID-19 has] made it worse for some people, and then they're not accessing services. And it's also true that we have a lot of families and individuals that have undiagnosed mental illness, and so those behaviors are exacerbated, absolutely.” ^*^Carla, behavioral health clinician
“I've seen rates of domestic violence go extremely up, and depression, and just overall anxiety…And so I think that has been affecting everyone” Sarah, behavioral health clinician
“Like many immigrant communities, they thrive on community, right? And the socialization and sharing of meals is so much a part of who they are, and what supports them, that the fact that they're being asked to socially isolate and wear masks where you can't see affect is a huge, huge kick […] for this population” Karen, behavioral health clinician
“....that would mean that just not having access to the information means that like, in addition to being like, physically isolated often, there is this like, emotional isolation as well, where people are like, ‘I'm here alone, and nobody can help me. And I don't necessarily know, in this new country how to help myself.' And that's not true, but I think a lot of people are put into a situation where they might feel that way.” William, case manager
**Financial stressors**
“I think that it's impacted them not only because of their health, but I think I said this before... they have lost jobs because if they were, for example, in the service industry, the restaurants are closed, or industrial services, cleaning services, any kind of service, that is again, frontline is more susceptible perhaps to not even being in existence anymore. So they don't have jobs or their hours have been cut. Or they have children that need to be taken care of and they don't have childcare” Zulma, behavioral health clinician
“That's [...] aside, but the financial, so they lose their jobs that they're already working in under the table. And they don't have, they don't have one of these two, they can't access money… They're not, they can't get unemployment, they can't get any kind of benefits, right. Any kind of assistance” Claudia, behavioral health clinician
**Preoccupation regarding transnational lives (*****n*** **=** **3; 14%)**
“I know, especially for folks who are living here and have family in Central America or have family in places where medical access is just – I mean, we maxed out our medical system in Guatemala in March. So, I know folks here [US] with family that are extremely fearful and scared of what will happen if someone gets COVID there [country of origin]. So yeah, I think those are the main challenges I've seen.” Sarah, behavioral health clinician
“And a lot of these families are also thinking about their families back home and sending money back home. So not only can you not provide for yourself and your immediate family that's with you, but you also can't provide for your family that are, you know, in whatever country [...] you're originally from” Carla, behavioral health clinician
“There were families that they work here in the US and send money back home, right... They're not.. they're not making money, right? So, when we talk about a challenge to one's identity, that's the big one, you're not able to provide, not just for your immediate family, you're not able to provide for the family that's back home.” Mateo, behavioral health clinician
**Secondary needs becoming more salient (*****n*** **=** **9; 43%)**
“In fact, with some of the parents, I can't talk about everything, because even if you ask someone: is this a privacy issue and they consent, right, like you asked me, [...] well I'm not gonna bring that in, because it's irrelevant, but I asked a mother [...] ‘how are things going?' She ended up emailing me later and saying, ‘even though I said that I had privacy to talk, I was unable to talk because my aunt was in earshot and we're staying with her, and my answers would have upset her.”' Karen, behavioral health clinician
“There just wasn't any support whatsoever. Again, access is the big one to technology. Again, right? Oh, you know, these internet companies were giving internet [...] free for three months, but then you were a customer after that, right? Well, caregivers who couldn't afford it before, can't afford it now. Technology again, it's a big one. How do you do remote work? If you have, you know, four or five other kids in the home, it's a single bedroom in a shared apartment. Internet is running slow.” Mateo, behavioral health clinician
**Immigration status as a main driver of inequality (*****n*** **=** **17; 81%)**
“Yeah, exactly, [...] this whole, like work from home thing really sticks out to me, because it's always been this place, this place of privilege. And I think even as a social worker, I never experienced, I never thought I would have a job where I could work from home. But I just know, in all of my clients' experience, that's just not possible. And if they don't work, they don't eat, and their kids don't eat. And so, I just don't think it was taken in consideration, especially in terms of, like stimulus packages, and how that affects undocumented folks.” Sarah, behavioral health clinician
“Now, these families are struggling 10 times more than the, you know, regular US population, who are documented and who can seek these services without feeling the fear of deportation” Camila, behavioral health clinician

* All the names provided are pseudonyms that have been created to protect the identity of participants.

#### Exacerbation of mental health symptoms

Most providers (*n* = 19; 90%) agreed that they noticed an exacerbation of mental health symptoms among Latinx parents and youth during the pandemic. Many of these mental health symptoms previously existed, such as depression, anxiety, post-traumatic stress disorder, domestic violence, alcohol misuse, panic attacks, and general feelings of stress; however, they were all exacerbated to higher levels of symptoms or experienced in a higher frequency. Providers mentioned that their clients were very concerned about working under risky conditions without the proper protection, contracting COVID-19, and losing their jobs or having their hours cut. Also, providers mentioned that, particularly among undocumented Latinx immigrants, there was much fear around contracting COVID-19 because then they would have to go to the hospital which could lead to deportation. Several providers (*n* = 8; 38%) reported that community support and ties were a vital support system and method of resilience among the Latinx community. However, during the pandemic, social distancing and other COVID-19 restrictions resulted in isolation from family and friends, negatively affecting their mental health. This included being unable to attend church, see family members, or attend community or social events. One of the providers mentioned the importance and sense of community that stems from socialization stating “being asked to socially isolate and wear masks where you can't see affect is a huge, huge kick […] for this population.”

#### Financial stressors

Providers (*n* = 18; 86%) indicated that one of the main stressors experienced by the Latinx community during the pandemic was related to finances. Providers indicated that most Latinx individuals experienced the loss of employment, cut hours at work, became essential workers, but obtained no extra pay to work in dangerous and often unhealthy conditions. Providers mentioned that finances were a huge stress, particularly because their clients were usually living pay check to pay check; therefore, losing hours or losing a job meant that several of their clients could no longer afford to pay rent or food. This lack of ability to provide was, in turn, generating a lot of anxiety in the Latinx immigrant clients who had migrated to the U.S. in search of better opportunities, but they were finding themselves in situations they might have experienced prior to migration. Providers also mentioned that some of their clients were afraid of receiving financial help or services they qualified for, such as food from their children's schools, because of fear stating, “they're already working in under the table…they can't access money…they can't get any kind of… assistance.”

#### Preoccupation regarding transnational lives

The providers interviewed indicated that the financial stressors were also tied to the transnational lives of their clients. A few providers (*n* = 3; 14%) indicated that their clients' mental health was also being impacted by their transnational ties in two main ways. On the one hand, providers felt that many of their Latinx clients were highly worried about the health and wellbeing of their family members and relatives back in their country of origin. On the other hand, providers expressed that some of their Latinx clients were worried about not being able to send remittances back home, which compounded the financial stressors they were already facing. Providers discussed this topic in-depth sharing that “a lot of these families are also thinking about their families back home and sending money back home.”

#### Secondary needs becoming more salient

Several providers (*n* = 9; 43%) identified that beyond basic necessities like food and water, the pandemic generated a situation in which prior secondary needs became basic, everyday necessities, particularly when trying to ensure that Latinx communities had access to services including a school for children and telehealth therapy. These secondary needs included reliable access to the internet and privacy in the home. Without these needs being covered, Latinx families were having a more difficult time accessing services, such as basic “internet” for “telehealth,” for their children and themselves.

#### Immigration status as a main driver of inequality

Most providers (*n* = 17; 81%) asserted that immigration status played a crucial role in how the Latinx community was able to deal with the consequences of the pandemic. Providers mentioned that undocumented immigrants were even more susceptible to the effects of the pandemic because they could not access government support like stimulus checks and/or unemployment assistance. Even when there was financial help available from private donors, making undocumented immigrants eligible for it, the funds usually ran out within the first hour, leaving several people unable to obtain any relief. Providers also mentioned that some of their undocumented clients could not provide proof of income, or did not have an official lease agreement, making them ineligible for emergency financial assistance or rent relief.

Providers mentioned that for their Latinx undocumented clients, not accessing any safety net during this time made them feel excluded, forgotten, and it made them question their belonging in the country. Providers also noted that the hostile rhetoric used by the administration in charge during the pandemic further exacerbated feelings of stress and hopelessness. One provider shared how “these families are struggling 10 times more than the, you know, regular US population, who are documented and who can seek these services without feeling the fear of deportation.”

## Discussion

Qualitative interviews with direct service providers to Latinx communities around mental health needs during COVID-19 identified five themes that summarized the difficulties faced by Latinx immigrant families faced during these unprecedented times in contemporary history.

Study findings reinforce literature on the intersectional issues faced by Latinx individuals related to their marginalized identities, including poverty, language barriers, lack of health insurance, and immigration status (25). During the pandemic, the Latinx immigrant population was further marginalized by the lack of policies that focused on their specific needs. According to providers, the COVID-19 pandemic exacerbated mental health symptoms among the Latinx immigrant population, and the mental health consequences of the pandemic will likely linger for many years (26). Therefore, it is important for health and mental health providers to monitor and screen patients using validated screenings coupled with culturally grounded conversations around mental health and wellbeing (27).

The exacerbation of mental health symptoms described by study participants is consistent with the COVID-19 Latinx mental health disparities literature (26). While the research on Latinx children is limited, Blanco et al. (28) found that social distancing for Latinx children living in California (e.g., social distance learning) was another source of stress. These findings suggest that it is critical for mental health providers to focus on the Latinx immigrant community and address mental health symptoms through increased mental health access and an increased number of mental health practitioners who are culturally and linguistically competent to meet their mental health needs. It is important for health and mental health providers to pay particular attention to signs of distress among the Latinx immigrant population and try to establish conversations around the stressors the pandemic brought about and how they have been coping with them. Now that pre-pandemic activities are resuming, providers need to reach out to Latinx families and other families who had a particularly difficult time during the pandemic to ensure their mental health needs are being covered.

The study findings around financial stressors align with previous studies indicating lost income (28, 29) and financial hardships, with greater increases among families with undocumented immigrants (30). Under these circumstances, mental health providers often address basic needs, rather than focusing on treating mental health symptoms. Given the changing world that we live in, it is necessary to understand that what used to be considered “basic necessities” have fluctuated as the world has adapted to the pandemic to include internet and privacy. This indicates that changes need to occur at the policy and institutional levels to ensure the holistic wellbeing of Latinx immigrant families, regardless of legal status. Also, providers need know about resources that immigrant families can apply for without fear of being considered a public charge their fears around applying for resources. At the institutional level, health centers need to offer integrated care, where the clients' basic needs including housing, food, and utilities, can be addressed in conjunction with their physical and mental health needs.

This study also describes how Latinx families were worried about the lives of their families in their country of origin with our findings differing from what previous studies have shown. Several studies have found that remittance decrease the likelihood of psychological distress (31) and depression (32). However, our results need to be considered within COVID-19, where 69% of Latinxs with a foreign-born family member lost jobs, worked fewer hours, or lost income, and 54% indicated substantial material hardship due to the pandemic (33). Given the economic impact of COVID-19, it is possible that the role of remittances changed while people worked toward economic stability. This finding highlights the importance for providers to explore the nature of transnational relationships when working with Latinx immigrants to identify stressors and their impact on mental health. Transnational relationships present an essential opportunity for providers to discuss with their clients during therapeutic encounters to ensure that these relationships remain a positive source of support and strength for Latinx immigrants, rather than an additional stressor to their daily lives.

Finally, according to our participants, immigration status was the main driver of inequity during the COVID-19 pandemic. Legal status became a determining factor in whether Latinx immigrants had access to necessary aid. For instance, families without documentation were ineligible for unemployment benefits and stimulus checks, leaving those who lost their jobs without aid (20). At the same time, those who could maintain their jobs and were deemed essential workers were at a higher risk of contracting COVID-19 and putting their families at risk while still not experiencing the same employment or health protections as Latinxs with legal status (20). The intersectional identities of Latinx immigrants, particularly of undocumented immigrants, exacerbated the needs they faced during the COVID-19 pandemic. Providers can play a key role in identifying and discussing how these intersectional identities impact clients differently. As the pandemic continues to uncover and exacerbate disparities, more resources are needed to support the Latinx population, particularly immigrants who lack documentation.

### Limitations

The generalizability of study findings is limited by the sample of providers working with low-income Latinx immigrant families. We acknowledge that the Latinx diaspora is quite diverse, and Latinx immigrants come from different countries and socio-economic backgrounds. Second, responses from different types of providers (e.g., mental health, health) were analyzed together; however, it is possible that other themes would have arisen if analysis had been stratified by provider type. Despite its limitations, the study is novel in its qualitative examination of mental health needs of Latinx immigrant communities from the perspectives of providers during an ongoing pandemic.

## Conclusion

Our findings from direct service providers highlight the detrimental mental health effect of COVID-19 in Latinx immigrant communities. It is essential to address the mental health needs of this population and ensure their equitable access to mental health services. Telehealth holds promise for Latinx clients and should be offered to meet the crucial mental health needs of this community and reduce systemic barriers to access and care.

## Data availability statement

Data will be made available upon request to the corresponding author.

## Ethics statement

Boston College Institutional Review Board authorized all procedures under Protocol Number 21.040.01e. The patients/participants provided their written informed consent to participate in this study.

## Author contributions

MP-L: conceptualization, methodology, formal analysis, investigation, writing—original draft, and visualization. NJP-F, KD, and KR: methodology, formal analysis, investigation, writing—original draft, and writing—review and editing. GO: writing—review and editing. SS: conceptualization, writing, visualization, senior review, and editing. All authors contributed to the article and approved the submitted version.

## Funding

This study was partly funded by the Center for Social Innovation at Boston College School of Social Work. MP-L was supported by the National Institute of Minority Health Disparities (R01 MD014694-04S1).

## Conflict of interest

The authors declare that the research was conducted in the absence of any commercial or financial relationships that could be construed as a potential conflict of interest.

## Publisher's note

All claims expressed in this article are solely those of the authors and do not necessarily represent those of their affiliated organizations, or those of the publisher, the editors and the reviewers. Any product that may be evaluated in this article, or claim that may be made by its manufacturer, is not guaranteed or endorsed by the publisher.
