# Supplementary material for: Mental health disparities in Latinx immigrant communities residing in the United States during COVID-19: Implications for policy and practice
Source: Front Public Health. 2022 Sep 29;10:1000233. doi: 10.3389/fpubh.2022.1000233 (PMC9558270; doi:10.3389/fpubh.2022.1000233)
Supplement: Supplementary file 1 [file Data_Sheet_1.docx]

**Electronic Supplemental Material: Interview guide**

**The COVID-19 pandemic [has] changed many people’s lives since it began. I’d like to ask you a few questions about if and how it has affected Latinx immigrants in the US.**

1. How do you feel the pandemic has affected Latinx immigrants?
   1. Sub-questions: Has it affected their ability to make money? Living situation? Relationships? Ability to access basic life necessities like food, medical care, child care, education, or other?
2. Have the needs of Latinx immigrants changed since the pandemic began?
3. Has your organization continued to provide all services since the pandemic began?
   1. Sub-questions: Which services and how has service delivery changed? What about other organizations who are directly serving migrants?
4. Are there services you wish you could provide but are [were] unable to due to the pandemic? What is preventing your organization from providing these services?
   1. Sub-questions: Where are [were] people going or what are [were] they doing to get these services and address other unmet needs?
5. In your opinion, has the pandemic further affected the mental health of Latinx immigrants? If so, how?
6. Do you think the situation of recent Latinx immigrants has been adequately considered during the COVID-19 crisis? If so, tell me what you think is going right. If not, tell me what you think is going wrong or missing from the response.
